# Short-term outcomes in patients with center-involving diabetic macular edema after a single dose of intravitreal bevacizumab

**DOI:** 10.1186/s40942-022-00430-z

**Published:** 2022-11-17

**Authors:** Christopher A. Turski, Mitchell A. Jacobs, Michelle M. Abou-Jaoude, Nicholas H. Fowler, Ryan Harpole, Emily Altman, John B. Chadwell, Gabriel Kindl, Hayley R. James, Shivani V. Reddy, Ramiro S. Maldonado

**Affiliations:** 1 Department of Ophthalmology, University of Kentucky, Lexington, KY USA; 2 Department of Ophthalmology, Duke University Eye Center, 2351 Erwin Rd, Durham, NC 27710 USA

**Keywords:** Anti-VEGF, Bevacizumab, Center-involving diabetic macular edema, Central subfield thickness, Responders, Non-responders, Subretinal fluid

## Abstract

**Background:**

A significant portion of diabetic macular edema (DME) is refractory to anti-vascular endothelial growth factor (anti-VEGF) agents. This study investigates morphological and functional outcomes to a single intravitreal bevacizumab (IVB) injection in patients with center-involving DME (ciDME) at 4–6 weeks and compares treatment responders and non-responders based on spectral domain optical coherence tomography (SD-OCT) features.

**Methods:**

IRB approved observational, retrospective chart review of patients with ciDME, identified by ICD-10 code, who received IVB and underwent baseline and 4–6 weeks follow-up SD-OCT imaging between January 1, 2016 and January 19, 2021. Patients who had received previous treatment with anti-VEGF or intraocular steroids within 1 year were excluded. Variables included best-corrected visual acuity (BCVA), central subfield thickness (CST) and total macular volume (TMV). Eyes were classified as responders if CST reduction was greater than 10%. OCT scans were graded qualitatively by two masked graders using Imagivault software. Paired Student’s t-tests, Wilcoxon signed rank tests and Chi-Square tests were used for analysis.

**Results:**

A total of 334 prospective subjects were identified, and after applying exclusion criteria 52 eyes from 46 patients (mean age 64.22 ± 8.12 years, 58.7% male) were included. Mean BCVA did not significantly change with treatment, 63.9 ETDRS letters (~ 20/50) at baseline and 65.9 ETDRS letters (~ 20/50) post-treatment (p = 0.07). Mean CST decreased from 466 ± 123 μm at baseline to 402 ± 86 μm post-treatment (p < 0.001). 22 (42.3%) of eyes were categorized as responders and 30 (57.7%) as non-responders. Average change in CST from baseline in responders was -164 μm (p < 0.001) and + 9 μm in non-responders (p = 0.47). Vitreomacular adhesion (VMA) was more prevalent in non-responders (28.7% vs. 4.8%, p = 0.03). In addition, cyst location in the inner nuclear layer (INL) was present more frequently in responders (95.5% vs. 73.3%, p = 0.037) as was subretinal fluid (45.5% vs. 13.3%, p = 0.01).

**Conclusion:**

The short-term response to a single IVB was sub-optimal with structural but no functional improvements. Greater baseline CST, presence of INL cysts and subretinal fluid may represent factors indicative of a better treatment response.

## Background

Diabetic macular edema (DME) represents a common vision-threatening complication of diabetes mellitus (DM) [1, 2]. It has been estimated that from disease onset, approximately 27% of patients with type 1 DM over 9 years and 28% with type 2 DM over 20 years will develop DME [3, 4]. Intravitreal injections of anti-vascular endothelial growth factor (anti-VEGF) inhibitors have become the first-line treatment in patients with center-involving DME (ciDME) coupled with rigorous metabolic control [5, 6]. Patients with ciDME routinely undergo multiple intravitreal injections of anti-VEGF over extended time intervals which places a considerable treatment burden on both patients and the health care system [7, 8]. It would therefore be beneficial to be able to anticipate what kind of initial response patients are likely to have to as early as possible.

Several algorithms and guidelines for the treatment of ciDME, based on anatomical and functional response to anti-VEGF, have been suggested [9–11]. Many of these algorithms center around spectral domain optical coherence tomography (SD-OCT), which has become integral to the diagnosis and monitoring of patients with DME through reproducible and high-resolution imaging of retinal anatomy. In addition to assessing retinal thickness and volume, SD-OCT has been used to evaluate qualitative features of DME such as the integrity of inner and outer retinal layers, hyper-reflective material and intraretinal cysts. Several studies have investigated whether these parameters may predict anatomical and functional outcomes in patients treated for DME, and consequently represent potential biomarkers of disease progression [12–16].

Improved understanding of such biomarkers may provide further insight into improving our care of patients with DME. A considerable number of patients do not respond to first-line anti-VEGF therapy, posing a significant unmet need in the management of DME [17]. It is therefore important to distinguish between responders and non-responders to be able to offer patients individualized treatment. Further, in elucidating biomarkers of non-responders, we may gain pathophysiological insight into potential avenues for new interventions.

The aim of this study was therefore to characterize the initial treatment response based on quantitative and qualitative OCT characteristics, in patients with ciDME receiving a single dose of intravitreal bevacizumab (IVB), in order to assist clinicians in their decision-making regarding future therapy.

## Materials and methods

The study was an IRB approved observational, retrospective study conducted at the Department of Ophthalmology at the University of Kentucky in Lexington, USA, which included data from patients with DME, identified by the International Classification of Diseases Code 10 (ICD-10), who received an IVB injection and underwent baseline and 4–6 weeks follow-up SD-OCT imaging between January 1, 2016 and January 19, 2021. Approval to review medical record data of the patients was obtained from the Institutional Review Board (IRB) of the University of Kentucky. All procedures adhered to the tenets of the Declaration of Helsinki.

Inclusion criteria were age > 18 years, diagnosis of Type 1 or Type 2 diabetes mellitus and ciDME. When both eyes of a patient met eligibility criteria, they were both included in the study. Exclusion criteria included previous treatment with anti-VEGF or intraocular steroids within 1 year, history of focal laser for macular edema or panretinal photocoagulation, history of vitreoretinal surgery or missing imaging. Medical records of each participant were reviewed for age, gender, duration of diabetes mellitus, and most recent glycated hemoglobin level at the baseline visit preceding IVB injection.

Best-corrected visual acuity (BCVA) at baseline and follow-up visits was measured with Snellen charts and converted to approximate Early Treatment Diabetic Retinopathy Study (ETDRS) letter equivalents for analysis. A SD-OCT device (Spectralis, Heidelberg Engineering, Heidelberg, Germany) was used to obtain volumetric macular scans with a scanning protocol of 20 × 20° and 25 OCT horizontal sections (one section every 240 μm). Central subfield thickness (CST) and total macular volume (TMV) were obtained at each study visit. Patients were divided into two subgroups based on reduction of CST encompassing responders (decrease by ≥ 10%) and non-responders (decrease by < 10%) [18, 19].

OCT images at baseline and follow-up visits were graded for qualitative features by two independent retina specialists (HRJ and SVR) who were masked to patient information. For cases in which a consensus result could not be reached, a third masked grader (CAT) made the final decision. Single central foveal OCT frames for each visit were graded, without image subdivisions, using the image grading software “Imagivault” (Melbourne, Australia).

The vitreoretinal interface was evaluated for the presence of vitreomacular adhesion (VMA), vitreomacular traction (VMT), posterior vitreous detachment (PVD), and epiretinal membrane (ERM) presence and severity (thick vs. thin). SD-OCT assessment also included presence and location of cysts (within ganglion cell layer, inner nuclear layer, outer plexiform layer and/or outer nuclear layer), shape of cysts (elongated, polygonal, round), and cyst reflectivity (hyporeflective, defined as similar to the vitreous; isoreflective, defined as similar to the inner nuclear layer; hyper-reflective, defined as similar to the retinal pigment epithelium; and mixed). Additional intraretinal features evaluated were the presence of hyper-reflective material (intracystic vs. non-cystic), hard exudates (five or more vs. less than five) defined by a size ≥ 100 μm, disorganization of retinal inner layers (DRIL) of at least 50 μm horizontal extent, and any disruption of the external limiting membrane (ELM) and ellipsoid zone (EZ).

Subretinal features analyzed included the presence of subretinal fluid (SRF) and SRF reflectivity (using the same reflectivity definitions as above).

Paired Student’s t-tests and Wilcoxon signed rank tests were used to compare quantitative OCT parameters in the entire study cohort and each subgroup before and after treatment. Chi-Square (χ^2^) tests were applied to analyze qualitative OCT parameters between subgroups at baseline. Select qualitative OCT features in the responder subgroup were further compared by means of McNemar tests before and after treatment. Shapiro–Wilk tests were used to assess normality of the data. Intergrader agreement was assessed with the Fleiss’ kappa (κ) statistic. Statistical analyses and graphical representation were performed using SPSS software version 28.0.0.0 (SPSS Inc., Chicago, Illinois, USA) and GraphPad Prism version 9.0.0 (GraphPad Software, San Diego, California, USA). Statistical significance was set at p < 0.05 and all tests were two-sided.

## Results

A total of 334 potential subjects with DME were identified by ICD-10 code. After applying inclusion and exclusion criteria, 58 eyes from 51 patients remained as the initial cohort. Following the grading process, 6 eyes were excluded from further evaluation due to poor image quality, resulting in a final cohort of 52 eyes from 46 patients (mean age 64.22 ± 8.12 years, 58.7% male) for final analysis. The mean duration of DM was 18.47 ± 9.92 years and the mean glycated hemoglobin level 8.05 ± 1.83%. Mean age, gender, ethnicity, duration of DM, most recent glycated hemoglobin level at baseline and diabetic retinopathy status are provided in Table 1.

**Table 1:** Patient baseline characteristics

	Cohort	Responders	Non-Responders	p-value
No. patients, n	46	19	27	
No. eyes, n	52	22	30	
Age, yr, mean ± SD	64.22 ± 8.12	63.61 ± 10.14	64.48 ± 6.79	0.3
Gender (M/F), n	(27/19)	(12/7)	(15/12)	0.6
Ethnicity, n (%)				
African American	9 (19.6)	3 (15.8)	6 (22.2)	0.58
Asian	0 (0)	0 (0)	0 (0)	
Hispanic	4 (8.7)	2 (10.5)	1 (3.7)	0.36
White	33 (71.7)	14 (73.7)	20 (74.1)	0.9
Diabetes mellitus (Type 1/Type 2), n	(0/46)	(0/19)	(0/27)	
Disease duration*, yr, mean ± SD	18.47 ± 9.92	20.55 ± 12.08	16.6 ± 7.66	0.6
HbA1C^**†**^, %, mean ± SD	8.05 ± 1.83	8.37 ± 2.26	7.82 ± 1.46	0.1
DR grade^§^, no. eyes (%)				
Mild NPDR	7 (16.6)	2 (11.8)	5 (20)	0.48
Moderate NPDR	11 (26.2)	4 (23.5)	7 (28)	0.75
Severe NPDR	13 (31)	8 (47)	5 (20)	0.06
PDR	11 (26.2)	3 (17.7)	8 (32)	0.3
BCVA (ETDRS letters), mean	63.9	60.9	66	0.09
CST (μm), mean ± SD	466 ± 123	560 ± 96	397 ± 90	< 0.001

HbA1C: Glycated hemoglobin level; DR: Diabetic retinopathy; NPDR: Non-proliferative diabetic retinopathy; PDR: Proliferative diabetic retinopathy; BCVA: Best-corrected visual acuity; ETDRS: Early Treatment Diabetic Retinopathy Study; CST: Central subfield thickness

Data are presented as number (%) with p-values between responders and non-responders derived from respective Mann Whitney U tests and Chi-Square (χ^2^) analyses.

*Data included from 19 patients; ^**†**^Data included from 37 patients; ^§^Data included from 42 eyes

**Fig. 1:**
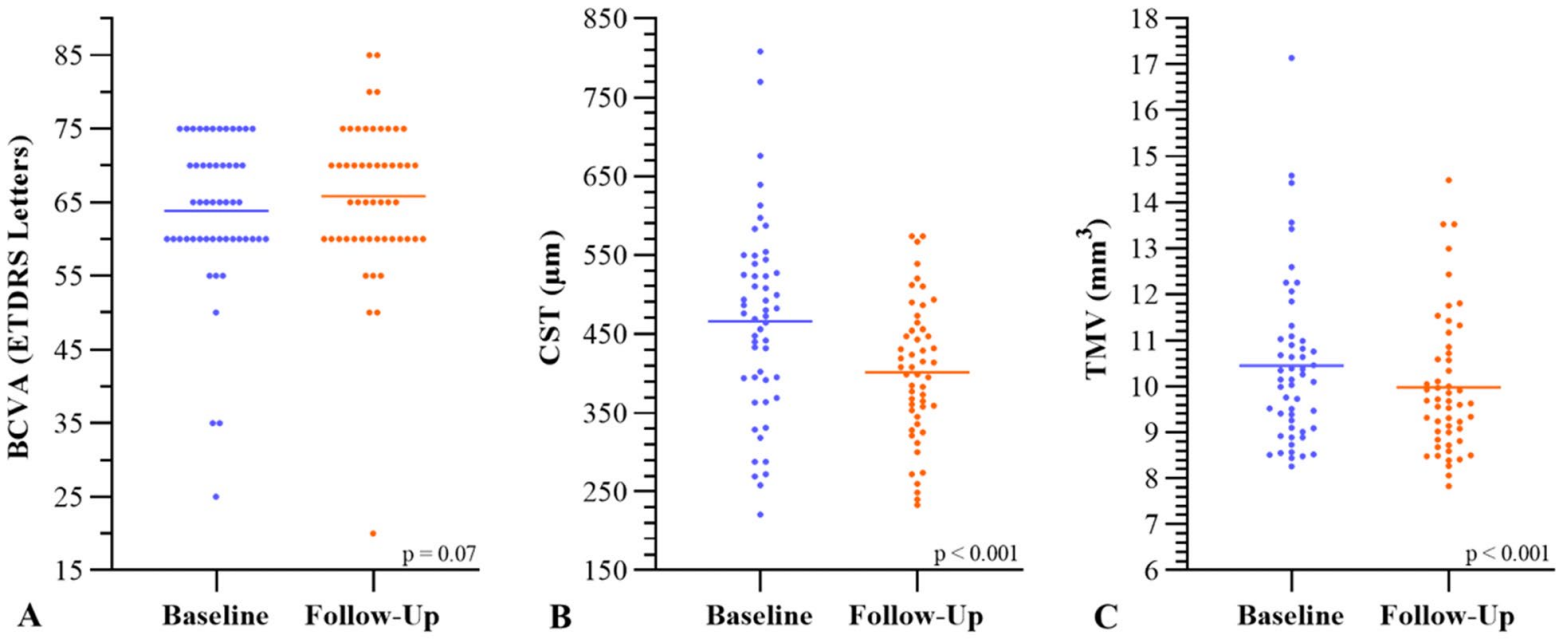
Scatterplots of BCVA and OCT metrics at baseline and follow-up in the entire study cohort. The cohort included 52 eyes. **A** Best-corrected visual acuity. **B** Central subfield thickness. **C** Total macular volume. Blue and orange horizontal lines indicate mean and standard deviation. P-values were derived from paired Student’s t-tests and Wilcoxon signed rank tests. Significant at p < 0.05

Mean BCVA did not significantly change from 63.9 ETDRS letters (Snellen equivalent, 20/50) at baseline to 65.9 ETDRS letters post-treatment (Snellen equivalent, 20/50) (p = 0.07). There was an improvement of ≥ 2 Snellen lines in 10 (19.2%) of eyes, with 4 (7.7%) of eyes improving by ≥ 3 Snellen lines. Mean CST decreased from 466 ± 123 μm at baseline to 402 ± 86 μm after treatment (p < 0.001) and a reduction in TMV was detected from 10.45 ± 1.8 mm^3^ at baseline to 9.98 ± 1.5 mm^3^ after treatment (p < 0.001) (Fig. 1).

**Fig. 2:**
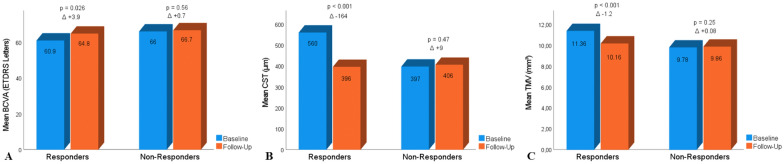
Column charts illustrating mean change in BCVA and OCT metrics from baseline in response subgroups. The responder subgroup included 22 eyes and the non-responder subgroup 30 eyes. **A** Best-corrected visual acuity. **B** Central subfield thickness. **C** Total macular volume. P-values were derived from paired Student’s t-tests and Wilcoxon signed rank tests. Significant at p < 0.05

When applying CST reduction metrics, 22 (42.3%) of eyes were categorized as responders (≥ 10% reduction) and 30 (57.7%) as non-responders (< 10% reduction). Mean BCVA improved from baseline in responders by + 3.9 ETDRS letters (p = 0.026) and in non-responders by + 0.7 ETDRS letters (p = 0.56). Average change in CST from baseline in responders was − 164 μm (p < 0.001) and in non-responders + 9 μm (p = 0.47). Furthermore, mean change in TMV from baseline in responders was − 1.2 mm^3^ (p < 0.001) and in non-responders + 0.08 mm^3^ (p = 0.25) (Fig. 2).

Intergrader agreement for the assessed qualitative OCT features, described by the κ statistic, ranged from 0.23 to 0.78. Highest values were achieved for the assessment of SRF (78%) and SRF reflectivity status (75%) (Table 2). The qualitative OCT characteristics of the treatment response groups are presented in Table 3. When assessing the vitreoretinal interface, VMA was less prevalent in responders than in non-responders (4.8% vs. 28.7%, p = 0.03) and PVD more prevalent in responders than in non-responders (85.6% vs. 60.7%, p = 0.055). A higher occurrence of INL cysts in responders (95.5% vs. 73.3%, p = 0.037) was observed. There was no statistically significant difference between the presence of DRIL (45.5% vs. 33.3%, p = 0.38), ELM disruption (13.6% vs. 13.3%, p = 0.98) or EZ disruption (36.4% vs. 26.7%, p = 0.45) between the subgroups.

**Table 2:** Intergrader agreements for qualitative OCT features from greatest to least

OCT feature	Kappa value
SRF	0.78
SRF reflectivity	0.75
IHRF (intracystic)	0.57
EZ disruption	0.56
ERM	0.47
ERM severity	0.46
Vitreous status	0.45
Exudates	0.44
Intraretinal cysts	0.44
IHRF (non-cystic)	0.42
ELM disruption	0.33
DRIL	0.32
Cyst reflectivity	0.23

OCT: Optical coherence tomography; SRF: Subretinal fluid; IHRF: Intraretinal hyper-reflective foci; EZ, Ellipsoid zone; ERM: Epiretinal membrane; ELM, External limiting membrane; DRIL: Disorganization of retinal inner layers

Assessed with the Fleiss’ kappa (κ) statistic

Strength of agreement was determined by the following scale: Poor < 0.20; Fair 0.21–0.40; Moderate 0.41–0.60; Good 0.61–0.80; Very good 0.81–1.00

**Table 3:** OCT characteristics at baseline for morphological responders and non-responders to one intravitreal bevacizumab injection

	Responders (n = 22)	Non-responders (n = 30)	p-value
Vitreous status, n (%)			
Completely attached	1 (4.8)	1 (3.6)	0.8
VMA	1 (4.8)	8 (28.7)	0.03
VMT	1 (4.8)	2 (7)	0.73
Completely detached	18 (85.6)	17 (60.7)	0.055
ERM, n (%)	14 (63.6)	13 (43.3)	0.15
Thick ERM	1 (7.1)	3 (23.1)	0.47
Thin ERM	13 (92.9)	10 (76.9)	0.065
Intraretinal cysts, n (%)			
Ganglion cell layer	5 (22.7)	4 (13.3)	0.38
Inner nuclear layer	21 (95.5)	22 (73.3)	0.037
Outer plexiform layer	2 (9.1)	4 (13.3)	0.64
Outer nuclear layer	22 (100)	25 (83.3)	0.07
Elongated shape	15 (68.2)	17 (56.7)	0.4
Polygonal shape	18 (81.8)	23 (76.7)	0.65
Round shape	12 (54.5)	10 (33.3)	0.13
Hyporeflective	8 (36.4)	6 (20)	0.19
Isoreflective	0 (0)	2 (6)	0.22
Hyper-reflective	0 (0)	0 (0)	
Mixed reflectivity	14 (63.6)	18 (60)	0.79
Hyper-reflective foci, n (%)	17 (77.3)	23 (76.7)	0.96
Intracystic	13 (59.1)	21 (70)	0.71
Non-cystic	16 (72.7)	21 (70)	0.83
Exudates, n (%)			
Absent	2 (9.1)	2 (6.7)	0.75
< 5 present	18 (81.8)	21 (70)	0.33
> 5 present	2 (9.1)	7 (23.3)	0.18
DRIL, n (%)			
No	12 (54.5)	20 (66.7)	0.38
Yes	10 (45.5)	10 (33.3)	
ELM disruption, n (%)			
No	19 (86.4)	26 (86.7)	0.98
Yes	3 (13.6)	4 (13.3)	
EZ disruption, n (%)			
No	14 (63.6)	22 (73.3)	0.45
Yes	8 (36.4)	8 (26.7)	
Subretinal fluid, n (%)			
No	12 (54.5)	26 (86.7)	0.01
Yes	10 (45.5)	4 (13.3)	
Hyporeflective	7 (70)	3 (75)	0.85
Isoreflective	0 (0)	0 (0)	
Hyper-reflective	0 (0)	0 (0)	
Mixed reflectivity	3 (30)	1 (25)	0.85

OCT: Optical coherence tomography; VMA: Vitreomacular adhesion; VMT: Vitreomacular traction; ERM: Epiretinal membrane; ELM: External limiting membrane; EZ: Ellipsoid zone; DRIL: Disorganization of retinal inner layers

Data are presented as number (%) with p-values derived from respective Chi-Square (χ^2^) analyses

Statistical significance was set at p < 0.05.

**Fig. 3:**
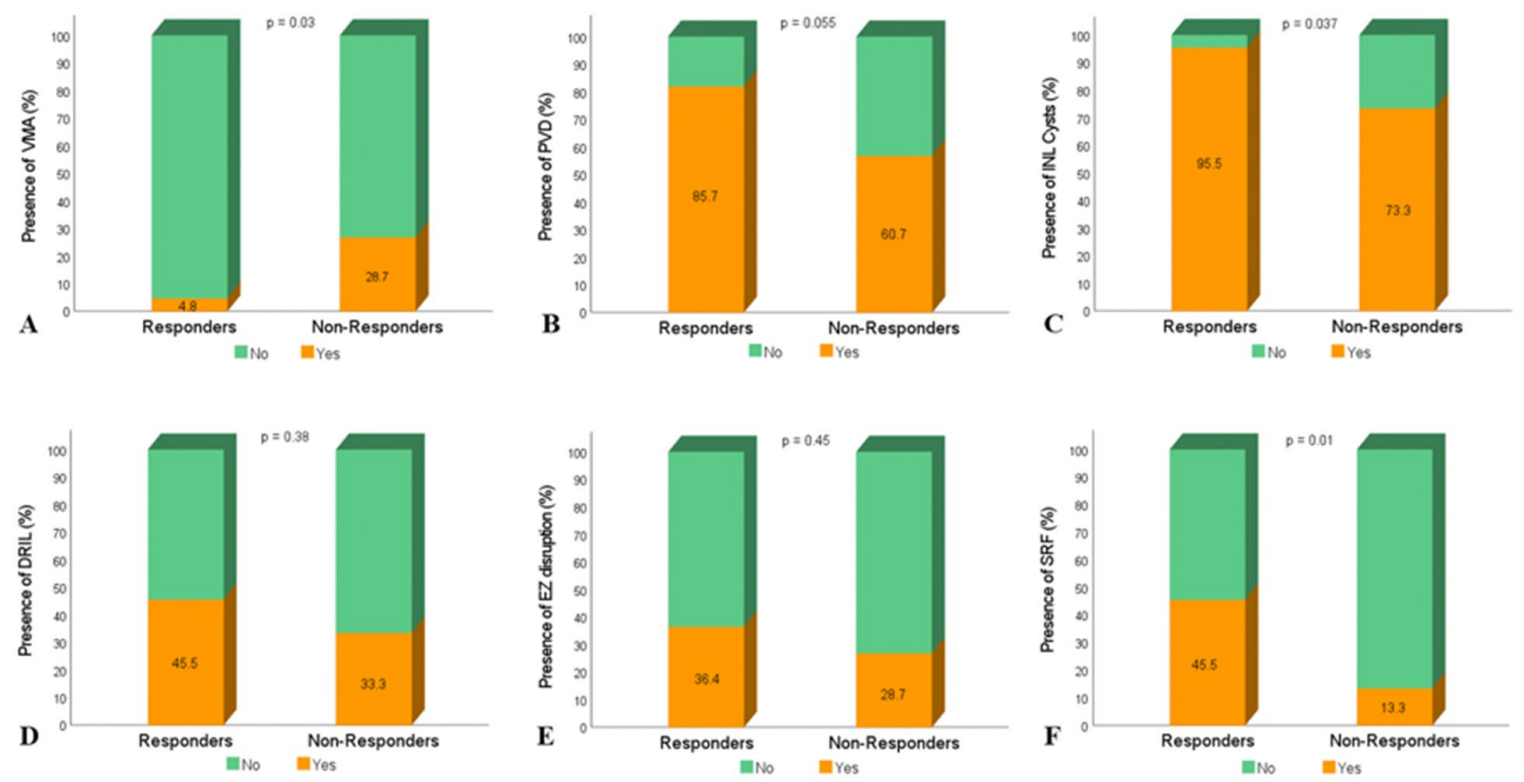
Column charts showing percentage of eyes graded with specific qualitative OCT characteristics at baseline. The responder subgroup included 22 eyes and the non-responder subgroup 30 eyes. **A** Presence of vitreomacular adhesion. **B** Presence of posterior vitreous detachment. **C** Presence of inner nuclear layer cysts. **D** Presence of disorganization of retinal inner layers. **E** Presence of disruption of ellipsoid zone. **F** Presence of subretinal fluid. P-values were derived from Chi-Square (χ^2^) tests. Significant at p < 0.05

**Fig. 4:**
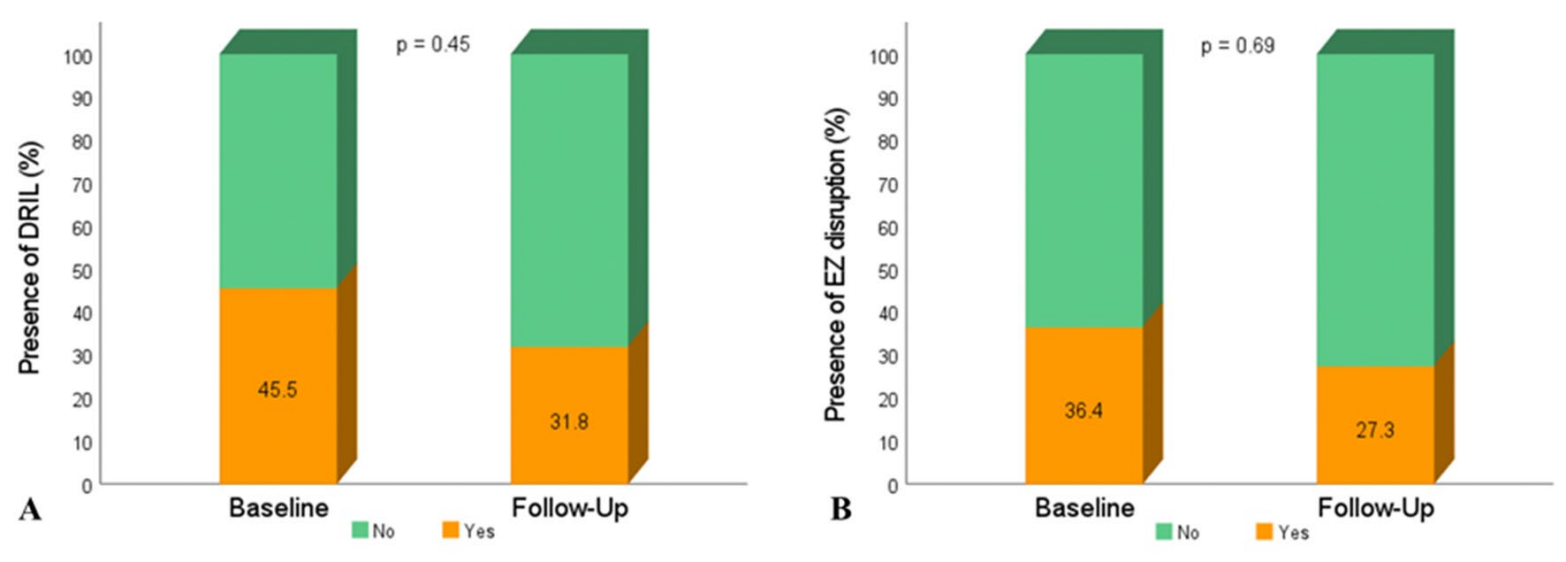
Column charts showing frequencies of qualitative OCT characteristics before and after treatment in responder subgroup. The responder subgroup included 22 eyes. **A** Presence of disorganization of retinal inner layers. **B** Presence of disruption of ellipsoid zone. P-values were derived from McNemar tests. Significant at p < 0.05

SRF was more prevalent in responders than in non-responders (45.5% vs. 13.3%, p = 0.01) (Fig. 3). When comparing the responder subgroup before and after treatment, there was a decrease in the presence of INL cysts (95.5% vs. 72.7%, p = 0.13), DRIL (45.5% vs. 31.8%, p = 0.45), EZ disruption (36.4% vs. 27.3%, p = 0.69) and SRF (45.5% vs. 31.8%, p = 0.45), which was not statistically significant (Fig. 4).

## Discussion

We present initial (4–6 weeks post-treatment) structural and functional analysis of ciDME response to IVB. In our study we show favorable structural response but no significant improvement in visual acuity. In addition, we found a high percentage of non-responders (57.7% not able to achieve more than 10% CST reduction). These non-responders had a higher prevalence of VMA and lower prevalence of PVD indicating a possible role of the vitreous-retina interface in response to treatment. Importantly, our study identified markers of response to treatment such as location of cysts in the INL and the presence of subfoveal fluid.

Improvement in visual acuity after one injection was uncommon as there were only 4 (7.7%) of eyes that gained ≥ 15 letters, and on average acuity was unchanged. This is in contrast to the 30–45% of patients with ≥ 15 letter gains after 1 year in major clinical trials such as RISE and RIDE or VIVID and VISTA [20, 21]. These findings highlight the need for multiple injections before a clinical effect is achieved. Our findings may provide clinicians with further data to discuss with patients when explaining the need for multiple injections. Promisingly, despite the initial lack of BCVA improvement, anatomical markers such as CST and TMV did demonstrate significant improvement after treatment (Fig. 1B and C).

The majority of our subjects fell into the non-responder group (57.7% of eyes). This is based on a 10% CST reduction from baseline. We adopted the 10% mark as it has been utilized in some studies and because using a 20% threshold used by some clinical trials was not feasible given our rather small sample size. In fact, the percentage of non-responders, had we used the 20% threshold, would have been 75%. In our study, the mean change in CST after a single IVB was − 64 μm. In the Diabetic Retinopathy Clinical Research Network (DRCR.net) Protocol T Study, a similar trend of CST reduction after IVB was detected after 4 weeks, which at the 1-year visit was on average at − 101 μm [18]. Furthermore, 79 of 207 eyes (39.2%) treated with IVB showed < 10% CST reduction after 12 weeks and 74 of 203 eyes (36%) had a CST of ≤ 250 μm after 1 year of continuous treatment [22]. In addition, a post hoc analysis from data of the DRCR.net Protocol I Study showed that of 118 eyes with a limited early anatomical response at 12 weeks, 37 (31.4%) and 61 (51.7%) subsequently achieved a ≥ 20% CST reduction at 1 year and 3 years, respectively, and were labelled “slow/late responders” [23]. Therefore, a limited initial anatomical response to anti-VEGF therapy does not necessarily preclude a further decrease in CST with continued treatment [23]. These findings suggest that with continuous therapy and long-term follow-up, given a similar trend of initial CST reduction despite our small sample size, fewer subjects in our cohort may have been categorized as non-responders at a later stage.

As discussed previously, CST is a reliable quantitative indicator of macular edema severity. Many clinical studies have relied upon CST as one of the most important outcomes when evaluating the efficacy of anti-VEGF therapy for ciDME [18–22]. Nevertheless, conflicting results have been reported regarding the predictive value of CST for treatment response [24, 25]. On the one hand, Soheilian et al. described a subgroup of eyes treated with IVB with an initial CST of ≥ 350 μm, being associated with better anatomical and visual outcomes. They suggested that eyes with higher CST might be more responsive to fluid resorption, consequently resulting in improved functional outcome [24]. Alternatively, Mushtaq et al. have shown that eyes with an initial CST of > 400 μm had a better anatomical response after receiving IVB, however, no significant difference in BCVA improvement was detected when comparing with eyes with a lower CST at baseline [25]. Baseline CST has been shown to be a significant determinant of subsequent CST response to anti-VEGF therapy, and the greatest reductions in CST in DME occur in eyes with highest baseline CST [26]. Our study highlights that a good anatomical response was to be expected with a higher CST at baseline (mean CST of 560 μm in the responder group vs. 397 μm in the non-responder group). However, it has to be stressed that our cohort consisted predominantly of non-responders (57.7%) and that DM was poorly controlled. In addition, the fact that a lower baseline CST does not translate into a better anatomical response might be explained in part by a “floor effect” on the amount of CST reduction that is possible in eyes with mild retinal thickening [23]. Furthermore, while the correlation between CST/TMV and BCVA in eyes treated for ciDME has been investigated in previous studies providing differing results [27, 28], in our study, there was a statistically significant difference in mean BCVA before and after treatment in the responder subgroup. This strengthens evidence that anatomic changes translate into functional improvements.

In addition to CST, several qualitative OCT features have been investigated to better characterize ciDME, and to identify predictive factors of treatment response [12–16]. Notably, the role of VMA has been investigated in functional and anatomical outcomes in ciDME [29, 30]. Sadiq et al. found that the presence of VMA at baseline with subsequent PVD corresponded with a greater potential for improvement in BCVA in eyes with ciDME over a period of 6 months [30]. However, reduction in central retinal thickness did not differ significantly between eyes with or without VMA. Hypotheses explaining these findings included improved transvitreal oxygenation after PVD or removal of an increased reservoir of accumulating growth factor in the premacular hyaloid with subsequent PVD [30]. In addition, it has also been hypothesized that VMA may play a role in the pathogenesis and persistence of DME by a mechanical traction component, potentially exerting its maximum effect in the case of focal foveolar attachment [31]. Subsequent release of this mechanical traction may therefore also be regarded as a possible explanation [31]. In our study, the presence of VMA was indicative of a poor anatomical response and, interestingly, the responder subgroup had a higher PVD percentage at baseline compared to non-responders (85.6% vs. 60.7% p = 0.055), which may be explained by previously described hypotheses.

Other qualitative features such as the presence of DRIL, ELM disruption and EZ disruption have previously been characterized as predictive of poor visual improvement in eyes with ciDME [12–14]. Studies have found that these parameters were associated with worse BCVA at baseline and a lower likelihood of improvement after treatment [12–14]. In our cohort, the responder subgroup had a higher occurrence of DRIL, ELM disruption and EZ disruption at baseline, which was surprising but not statistically significant. Here, we must acknowledge the graders only had very fair to low agreement in grading these features and thus, our qualitative findings for these features cannot be used to elaborate conclusions.

The presence of intraretinal cysts, IHRF and SRF has also been studied as prognostic factors of anti-VEGF response in DME patients. The relationship between fluid volume within the INL and visual acuity has been recently investigated [32]. In our study, a significantly larger number of responders presented with INL cysts at baseline, and Tsuboi et al. found that an increased INL fluid volume was strongly associated with worse BCVA. This may be partially explained by the fact that fluid may disrupt the function of bipolar, amacrine and horizontal cells located within the INL, subsequently affecting the transmission of visual information from photoreceptors to ganglion cells [32]. It could be speculated, that when INL cysts are present, this OCT feature could be indicative of an early/acute stage of ciDME, potentially more responsive to anti-VEGF therapy and therefore detected more frequently in anatomical responders, who in our case, also had a worse baseline mean BCVA compared to non-responders (60.9 vs. 66 ETDRS letters).

IHRF and exudates have also been previously studied as possible predictors of treatment response. Hwang et al. have shown that a higher number of IHRF was observed on SD-OCT at baseline in both non-responders to IVB and, interestingly, in responders to intravitreal dexamethasone therapy, indicating an activated inflammatory process in the retina [15]. In our study cohort, however, no significant difference was found in the occurrence of IHRF and exudates between the subgroups at baseline.

Furthermore, responders showed a significantly higher prevalence of SRF at baseline compared to non-responders. In our cohort (n = 52) the prevalence of SRF was 26.9%, which is in accordance with other studies estimating a prevalence of 15–30% in DME [33]. It remains unclear, however, whether the presence of SRF gives an indication on whether a good treatment response to anti-VEGF therapy can be expected. A post hoc analysis of the RISE and RIDE studies had shown that the presence of SRF at baseline can predict good anatomical and functional outcomes and that sustained VEGF suppression is effective in its reduction [34]. On the other hand, another study by Giocanti-Aurégan et al. could not confirm SRF being a good prognostic indicator during DME treatment, failing to find better visual outcomes when comparing with eyes not featuring SRF. Nevertheless, they did demonstrate a good anatomical response in eyes with SRF during the initial treatment phase up to the third intravitreal injection [35]. Our findings further support the presence of SRF being indicative of a good initial anatomical treatment response.

Our study has limitations, namely the retrospective design, the small sample size within subgroups, the overall fair to moderate strength of intergrader agreement, and the fact that no CST cut-off was applied as an inclusion criterion, which might pose a challenge for drawing definitive conclusions. However, the latter was decided upon in order to best comply with a real-world clinical setting. A further limitation in our study is the lack of lens status or macular hypoperfusion data in our patients. These factors could influence visual acuity and thus, preclude visual acuity improvements after treatment. Our study strengths include masked grading of qualitative OCT features and the evaluation of the immediate effect of IVB at 4–6 weeks post-treatment, a timepoint frequently not reported in clinical trials.

## Conclusions

The anatomical response to IVB in our study cohort was sub-optimal with majority of patients being non-responders. While no functional improvements were noted, structural responses were detected and some predictive factors of response were absence of VMA, presence of PVD, cystoid spaces in the INL, presence of SRF and higher baseline CST and TMV. These findings may provide further guidance for the initial management of ciDME in clinical practice when considering IVB as first-line treatment.

## Data Availability

The data used and/or analyzed during the current study is available from the corresponding author on reasonable request.

## Abbreviations

DME: Diabetic macular edema
DM: Diabetes mellitus
ciDME: Center-involving diabetic macular edema
VEGF: Vascular endothelial growth factor
SD-OCT: Spectral domain optical coherence tomography
IVB: Intravitreal bevacizumab
ICD-10: International Classification of Diseases Code 10
IRB: Institutional Review Board
CST: Central subfield thickness
BCVA: Best-corrected visual acuity
ETDRS: Early Treatment Diabetic Retinopathy Study
TMV: Total macular volume
VMA: Vitreomacular adhesion
VMT: Vitreomacular traction
PVD: Posterior vitreous detachment
ERM: Epiretinal membrane
DRIL: Disorganization of retinal inner layers
ELM: External limiting membrane
EZ: Ellipsoid Zone
SRF: Subretinal fluid
